# Investigation Into the Risk Factors Related to In-stent Restenosis in Elderly Patients With Coronary Heart Disease and Type 2 Diabetes Within 2 Years After the First Drug-Eluting Stent Implantation

**DOI:** 10.3389/fcvm.2022.837330

**Published:** 2022-05-20

**Authors:** Ming Yi, Wen-hui Tang, Shuai Xu, Xiao Ke, Qiang Liu

**Affiliations:** ^1^Department of Clinical Medicine, University of South China, Hengyang, China; ^2^Department of Cardiology, Fuwai Hospital, Chinese Academy of Medical Sciences, Shenzhen Sun Yat-sen Cardiovascular Hospital, Shenzhen, China; ^3^Department of Cardiology, Liuyang Hospital of Traditional Chinese Medicine, Changsha, China; ^4^Department of Cardiology, Shenzhen Traditional Chinese Medicine Hospital, Shenzhen, China

**Keywords:** in-stent restenosis, drug-eluting stent, categorical principal component analysis, percutaneous coronary intervention, blood glucose level, HbA1c

## Abstract

**Background:**

The present study aims to explore risk factors related to in-stent restenosis (ISR) in elderly patients with coronary heart disease and type 2 diabetes within 2 years after the first drug-eluting stent (DES) implantation.

**Methods:**

This case-control study retrospectively analyzed the clinical data of patients with coronary heart disease and diabetes undergoing percutaneous coronary intervention (PCI) in Shenzhen Sun Yat-sen Cardiovascular Hospital between January 2010 and March 2020. Univariate and multivariate models were used to assess independent factors for DES-ISR. Categorical principal component analysis of clinical variables was performed to determine important components for DES-ISR. Nomogram was constructed to quantitatively predict the probability of DES-ISR development. The diagnostic potential of clinical variables was determined by receiver operating characteristic curve.

**Results:**

In the derivation cohort, 1,741 cases were included in this study, and a total of 227 pairs of cases and controls were generated by propensity score matching. In the validation cohort, 102 cases were included with 19 cases (18.6%) with DES-ISR. Glomerular filtration rate <60 ml/min/1.73 m^2^, fasting blood glucose ≥6.5 mmol/L, multivessel coronary artery disease, coronary artery diffuse disease, PCI operation time (≥60 min), emergency PCI were associated with ISR. High Nomogram score was associated with the increased risk of ISR. Further analysis of the validation cohort showed that higher levels of HbA1c-coefficient of variation (CV) were significantly associated with the increased risk of ISR. HbA1c-CV exhibited good predictive ability for ISR in the validation cohort.

**Conclusions:**

In conclusion, the fasting blood glucose level during the perioperative period of emergency PCI and the long-term variation of HbA1c during the follow-up period are related to the incidence of DES-ISR and the degree of stenosis. Reducing blood glucose fluctuations may decrease the risk of DES-ISR.

## Introduction

Cardiovascular disease is the first major causes of deaths among all urban and rural residents in China according to China Cardiovascular Disease Report 2019, and coronary heart disease affects around 11 million people in China (1). Current prevention and treatment strategies for coronary heart disease mainly focus on primary prevention, improving follow-up, optimizing disease treatment strategies, and ultimately improving the prognosis of patients with coronary heart disease (2, 3). Coronary artery bypass grafting (CABG) and percutaneous coronary intervention (PCI) are the current standard treatments for coronary heart disease (4). PCI is a treatment option for patients with uncomplicated lesions, but its revascularization rate is significantly higher than that of CABG (5). Moreover, patients with diabetes who receive PCI treatment will have a higher incidence of major adverse cardiovascular events (MACEs) within 5 years and higher rate of repeated revascularization (6). However, due to advanced age, severe comorbidities (such as renal insufficiency, diabetes, etc.), complex coronary anatomy and reduced ejection fraction, more and more patients with coronary heart disease are at high risk of surgery. Therefore, PCI is usually provided to these high-risk patients to replace CABG (7).

In-stent restenosis (ISR) is an important cause of stent failure and repeated revascularization, and is the main limiting factor for PCI treatment (8). Although the use of drug-eluting stents (DES) reduces the occurrence of ISR when compared with bare-metal stents (BMS) (9), the prevalence of ISR remains relatively unchanged, accounting for about 3–20% of PCI treatment (10). According to the studies, age and diabetes are proven to be important predictors of ISR (11). Takao et al. (12), showed that long-term visit-to-visit variability in HbA1c and systolic blood pressure represented a combined and additive risk for cardiovascular disease incidence in patients with type 2 diabetes; Ceriello et al. (13), HbA1c variability was reduced by empagliflozin and high values of HbA1c variability were associated with an increased risk of cardiovascular death. Recently, Yang et al. (14), showed that HbA1c variability was associated with ISR in patients with type 2 diabetes after PCI; however, the exact mechanism of ISR is currently unclear. Therefore, strengthening the control of risk factors and clinical follow-up may be an important means to reduce the incidence of ISR. At present, the source of predicting ISR models constructed by screening risk factors includes patients implanted with BMS (11), and there is no risk factor prediction analysis for the special group of elderly patients with coronary heart disease and diabetes (15), so whether this population has more specific prediction method is worthy of further exploration. Therefore, we retrospectively analyzed the data of elderly patients with coronary heart disease and diabetes who underwent PCI intervention and implanted DES in Shenzhen Sun Yat-sen Cardiovascular Hospital from January 2010 to March 2020, and to explore the potential risk factors including glomerular filtration rate, fasting plasma glucose (FPG), multivessel coronary artery disease, coronary artery diffuse disease, PCI operation time, emergency PCI and HbA1c for ISR.

## Materials and Methods

### Patients

This study included 1,843 patients (≥65 years old) with coronary heart disease and diabetes undergoing PCI in Shenzhen Sun Yat-sen Cardiovascular Hospital between January 2010 and March 2020. All patients provided written and informed consent. The institutional review boards at Shenzhen Sun Yat-sen Cardiovascular Hospital approved the present study.

The inclusion criteria were as follow: (1) PCI was conducted according to the Chinese Guidelines for Percutaneous Coronary Intervention (2016). (2) Diabetes was diagnosed according to the National guidelines for the prevention and control of diabetes in primary care (2018). (3) Patients received DES implantation. (4) Patients received PCI for the first time in Shenzhen Sun Yat-sen Cardiovascular Hospital. (5) The duration of follow-up ≥24 months. (6) During follow-up, ISR was diagnosed by coronary angiography (CAG) and requiring target lesions revascularization (TLR). The exclusion criteria were as follow: (1) Patients received PCI in other hospitals. (2) Patients received BMS implantation. (3) Patients received CABG or stent grafting during the follow-up period. (4) Patients received revascularization treatment due to stent fracture.

### Collection of Patients Data

All clinical data were collected from the follow-up database of patients in Shenzhen Sun Yat-sen Cardiovascular Hospital. The clinical data including the basic characteristics of the patient, past medical history, medication history, CAG characteristics, and first PCI characteristics. All patients were treated with a standardized PCI strategy. Oral aspirin (600 mg) combined with clopidogrel (300 mg) were administered before emergency PCI. Oral aspirin (100 mg/day) combined with clopidogrel (75 mg/day), or ticagrelor (90 mg twice daily) after emergency PCI and elective PCI were given to patient for >1 year. The target lesion revascularization is defined as the stent placement or balloon expansion at the site of the stent due to angina pectoris. ISR is defined as the stenosis of the stent segment vessel lumen ≥50% as confirmed by CAG (15, 16). All data are laboratory test results during perioperative evaluation and follow-up period.

### Propensity Score Matched and Grouping

In the derivation cohort, 1,741 patients with coronary heart disease and diabetes undergoing PCI in Shenzhen Sun Yat-sen Cardiovascular Hospital between January 2010 and January 2016 were included. In this cohort, 233 DES-ISR patients and 1,508 non-DES-ISR patients were included in the study. Propensity score matching (PSM; 1:1) was performed to control for potential bias. Propensity scores were calculated using a logistic regression model with access route as the dependent variable. Independent variables included age and gender. In order to prevent poor matches, the caliper was set as 0.03.

### Statistical Analysis

All the data analysis was performed using IBM SPSS software version 25.0 (SPSS, Inc., Chicago, IL, United States), and the Nomogram was constructed using R software version 4.0.2. Continuous variables were presented as mean ± standard deviation; comparisons were conducted by Student's *t*-test or Mann-Whitney *U*-test when group distributions were skewed. Categorical variables were presented as percentages and relative frequencies; comparisons were conducted using chi-square statistics or Fisher exact test as appropriate. Multivariable Cox proportional hazards regression models with adjustments for confounding factors were used to assess the associations of clinical parameters with MACEs. Correlation analysis was evaluated by Pearson Correlation analysis. Correlations between clinical variables were detected by non-linear categorical principal component analysis (CatPCA). The CatPCA benefits from optimal data scaling and is suitable for data recorded with uncertain units (16). Original variables were reduced to two principal components determined to be sufficiently robust by a Cronbach > level >0.70 for each. The areas under the receiver operating characteristic curve (ROC), sensitivity, specificity and 95% confidence interval (CI) were calculated to evaluate the predictive ability of different predictors. Kaplan–Meier survival curves were constructed to evaluate the incidence rate of MACEs among different groups. ^*^P < 0.05 was considered statistically significant.

## Results

### Baseline Clinical Characteristics

The study design was summarized as a flow chart in Supplementary Figure S1. In the derivation cohort, a total of 1,741 patients received PCI were included in the analysis, and 233 patients with DES-ISR (13.4%) were identified among the study population. For the unmatched grouping, a total of 233 DES-ISR patients were included. For the controls, the controls were randomly selected from the 1,508 non-DES-ISR patients. Subsequently, two control (non-DES-ISR) patients were matched to each DES-ISR patient. The medical history, demographics and indications for DES-ISR for all the patients were shown in Table 1. The rates of glomerular filtration rate (GFR) <60 ml/min/1.73 m^2^, dyslipidemia, multi-vessel coronary heart disease (CHD), diffuse CHD and emergency PCI, number and total length of implanted stent, operation duration for PCI were significantly higher, the rate of single-vessel CHD and minimum diameter of implanted stent was significantly lower in the DES-ISR group than that in the non-DES-ISR group (Table 1). No significant differences in other parameters between the two groups were detected. For the PSM grouping, a total of 227 non-DES-ISR and 227 DES-ISR patients were included in the comparison (Table 1). Comparison outcomes were similar to that from unmatched grouping (Table 1).

**Table 1:** Baseline clinical characteristics of the study population.

**Variables**	**Unmatched population**		***P-*value**	**PSM cohort**		***P-*value**
	**DES-ISR (*n* = 233)**	**Non-DES-ISR (*n* = 466)**		**DES-ISR (*n* = 227)**	**Non-DES-ISR (*n* = 227)**	
Age (years old)	69.7 ± 7.9	71.5 ± 6.2	0.074	70.3 ± 6.3	70.3 ± 6.3	0.853
Gender (Male)	151 (64.8%)	297 (63.7%)	0.780	156 (68.7%)	146 (64.3%)	0.320
Smoking	79 (33.9%)	141 (30.3%)	0.328	72 (31.7%)	78 (34.4%)	0.549
Alcohol intake	11 (4.7%)	18 (3.9%)	0.592	10 (4.47%)	11 (4.8%)	0.823
BMI (kg/m^2^)	25.3 ± 1.9	22.4 ± 2.3	0.684	25.3 ± 1.9	22.5 ± 2.3	0.778
Heart rate (beats/min)	73.6 ± 14.2	73.9 ± 14.2	0.645	73.5 ± 14.3	73.8 ± 14.1	0.591
Systolic BP (mmHg)	139.1 ± 20.6	138.9 ± 2.6	0.161	138.6 ± 20.1	138.4 ± 21.5	0.408
Diastolic BP (mmHg)	77.8 ± 42.6	75.8 ± 12.5	0.309	77.6 ± 43.1	76.9 ± 12.7	0.533
GFR <60 ml/min/1.73 m^2^	19 (8.2%)	3(0.6%)	<0.001	19 (8.4%)	1 (0.4%)	<0.001
**Medical history**
Hypertension	190 (81.5%)	350 (75.1%)	0.056	182 (80.2%)	165 (72.7%)	0.060
Dyslipidemia	231 (99.1%)	175 (37.6%)	<0.001	226 (99.6%)	85 (37.4%)	<0.001
Family history of CHD	13 (5.6%)	15 (3.2%)	0.134	13 (5.7%)	6 (2.6%)	0.101
**Laboratory examinations**
Fasting blood glucose (mmol/L)	8.18 ± 4.84	7.25 ± 2.53	0.001	8.22 ± 4.89	7.30 ± 2.60	0.017
HbA1c (%)	7.19 ± 1.41	7.05 ± 3.10	0.388	7.20 ± 1.42	6.89 ± 1.56	0.871
LDL (mmol/L)	2.71 ± 1.05	2.51 ± 0.99	0.143	2.68 ± 1.04	2.52 ± 1.01	0.350
HDL (mmol/L)	1.11 ± 0.75	1.11 ± 0.67	0.697	1.11 ± 0.76	1.08 ± 0.23	0.173
Triglycerides (mmol/L)	1.75 ± 1.23	1.80 ± 1.91	0.259	1.75 ± 1.24	1.92 ± 2.45	0.087
**Medical treatment**
ACEI	130 (55.8%)	224 (48.1%)	0.054	128 (56.4%)	112 (49.6%)	0.145
ARB	58 (24.9%)	100 (21.5%)	0.306	54 (23.8%)	48 (21.1%)	0.500
Beta blockers	201 (86.3%)	375 (80.5%)	0.058	195 (85.9%)	184 (81.1%)	0.164
Statins	230 (99.6%)	458 (98.3%)	0.155	226 (99.6%)	223 (98.2%)	0.177
CCB	111 (47.6%)	197 (42.3%)	0.178	107 (47.1%)	87 (38.3%)	0.058
**Type of CHD**
Single-vessel	13 (5.6%)	166 (35.6%)	<0.001	13 (5.7%)	87 (38.3%)	<0.001
Multi-vessel	220 (94.4%)	300 (64.4%)	<0.001	214 (94.3%)	140 (61.7%)	<0.001
Diffuse	156 (67.0%)	267 (57.3%)	0.014	154 (67.8.5%)	131 (57.7%)	0.026
**Stent implantation**
Stent number	3.58 ± 1.87	2.12 ± 1.52	<0.001	3.50 ± 1.80	2.19 ± 1.84	0.005
Total length (mm)	80.2 ± 46.6	48.6 ± 30.2	<0.001	78.03 ± 43.6	49.21 ± 31.7	<0.001
Minimum diameter (mm)	2.57 ± 0.34	2.74 ± 0.63	0.048	2.58 ± 0.35	2.80 ± 0.81	0.046
**PCI**
Operation duration (min)	89.4 ± 39.3	69.9 ± 25.2	<0.001	89.85 ± 39.7	71.01 ± 25.8	<0.001
Type (emergency)	92 (39.5%)	92 (19.7%)	<0.001	89 (39.2%)	48 (21.1%)	<0.001

### Univariate and Multivariate Models for DES-ISR in the PSM Cohort

Univariate and multivariate models were used to assess independent factors for DES-ISR in the PSM cohort. The univariate analysis showed that clinical characteristics including GFR <60 ml/min/1.73 m^2^, dyslipidaemia, FPG, medium-chain acyl-CoA dehydrogenase (MCAD), PCI operation duration and type of PCI were significantly associated with the occurrence of DES-ISR in the PSM cohort (Table 2). The multivariate analysis further revealed that GFR <60 ml/min/1.73 m^2^, dyslipidaemia, FPG, MCAD, PCI operation duration and type of PCI were independent predictors for DES-ISR in the PSM cohort (Table 3).

**Table 2:** Univariate models for DES-ISR in the propensity matched population.

**Variables**	**OR**	**95% CI**	***P*-value**
GFR <60 ml/min/1.73 m^2^	2.76	1.40–5.46	0.004
Dyslipidemia	1.90	1.29–2.78	0.001
Fasting blood glucose (≥6.5 mmol/L)	4.80	2.29–10.06	<0.001
Multi-vessel CHD	7.30	3.13–17.02	<0.001
Diffuse CHD	1.59	0.82–3.08	0.168
Number of stent (≥2)	0.97	0.46–2.08	0.943
Total length of stent (≥45 mm)	1.18	0.42–3.33	0.757
Minimum diameter of stent (≤ 2.5 mm)	1.51	0.53–4.28	0.441
PCI operation duration (≥60 min)	2.62	1.12–6.13	0.027
PCI type (Emergency)	2.24	1.49–3.36	0.000

**Table 3:** Multivariate models for DES-ISR in the propensity matched population.

**Variables**	**β regression coefficient**	**OR**	**95% CI**	***P*-value**
GFR <60 ml/min/1.73 m^2^	1.019	2.77	1.41–5.47	0.003
Dyslipidemia	0.642	1.90	1.30–2.78	0.001
Fasting blood glucose (≥6.5 mmol/L)	1.705	5.50	3.05–9.92	<0.001
Multi-vessel CHD	1.982	7.26	3.27–16.11	0.000
Diffuse CHD	0.588	1.80	1.13–2.88	0.014
PCI operation duration (≥60 min)	0.963	2.62	2.13–6.05	0.024
PCI type (Emergency)	0.788	2.20	1.48–3.28	<0.001

### CATPCA Analysis of Clinical Variables in the PSM Cohort

**Figure 1:**
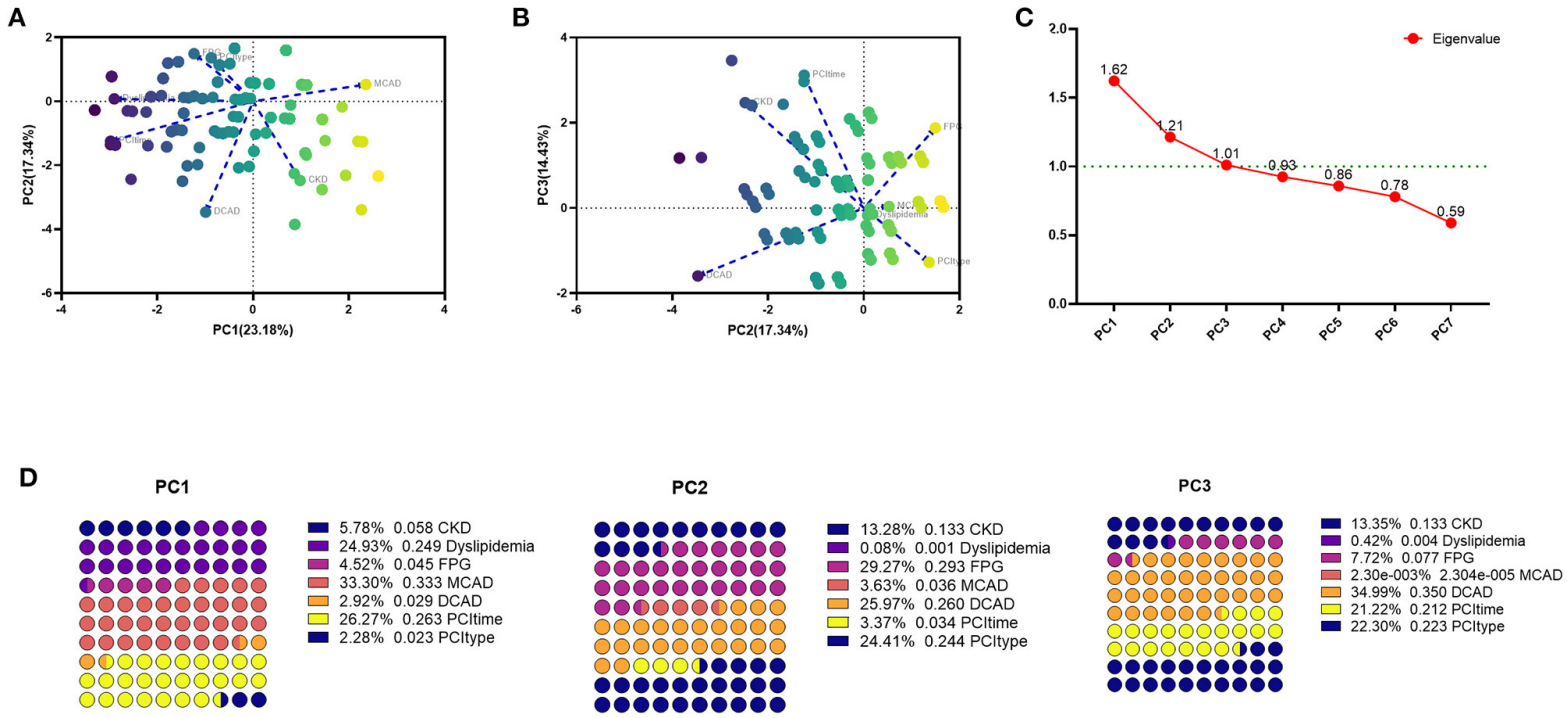
CATPCA analysis of clinical variables in the PSM cohort. **(A,B)** CATPCA biplot—component loadings of the most explanatory variables. **(C)** The scree plot of CATPCA. **(D)** Risk factors in PC1, PC2, and PC3.

Categorical principal component analysis of clinical variables was performed to determine important components for DES-ISR in the PSM cohort. Based on the CATPCA analysis, PC1, PC2, and PC3 explained 23.18, 17.34, and 14.43% of the total variation of the data, and these PCs explained more than 50% of the total variation of the data (Figures 1A–C). The most important components in PC1, PC2, and PC3 were illustrated in Figure 1D.

### The Association Between Nomogram Scores and DES-ISR

**Figure 2:**
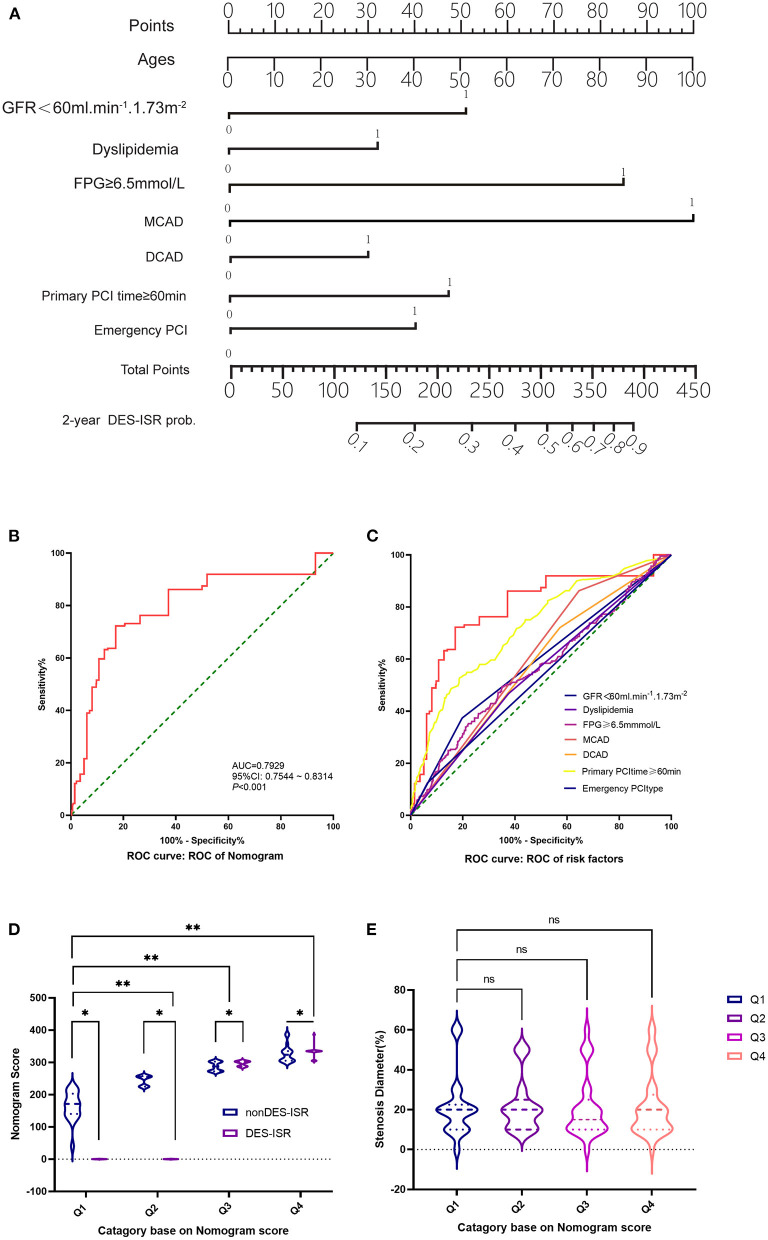
The association between Nomogram scores and DES-ISR. **(A)** Nomogram from the final multivariable analysis of the binary logistic regression model. **(B)** ROC curve and its diagnostic performance based on the Nomogram. **(C)** ROC curve and the diagnostic performance of individual risk factors. **(D)** The nomogram score analysis in non DES-ISR and DES-ISR patients. **(E)** The stenosis diameter (%) in patients with different category based on the nomogram score. ns = not significant, *P < 0.05, **P < 0.01.

In the derivation cohort, using the multivariate survival model, we constructed a nomogram to quantitatively predict the probability of DES-ISR development in the patients received PCI. The nomogram was based on GFR <60 ml/min/1.73 m^2^, dyslipidaemia, FPG, MCAD, PCI operation duration and type of PCI (Figure 2A). In the validation cohort, 102 patients with coronary heart disease and diabetes undergoing PCI in Shenzhen Sun Yat-sen Cardiovascular Hospital between January 2017 and March 2020 were included. In this cohort, 19 DES-ISR patients and 83 non-DES-ISR patients were included in the study. The predictive accuracy of the nomogram was assessed in an independent validation cohort (see Table 4 for the clinical parameters of the patients from the validation cohort) with a ROC analysis, showing good probability prediction for the prognostic multivariate model in the independent validation cohort (Figure 2B). The predictive accuracy of the nomogram was higher than that of individual variable (Figure 2C). Furthermore, the results also showed that the nomogram score in the DES-ISR group was significantly higher than that in the non-DES-ISR group. While, the nomogram score is not associated with SD (Figures 2D,E).

**Table 4:** Baseline clinical characteristics of derivation cohort and validation cohort.

**Variables**	**Derivation cohort (*n* = 454)**	**Validation cohort (*n* = 102)**	***P*-value**
Age (years old)	70.3 ± 6.3	71.4 ± 7.2	0.056
Gender (Male)	302 (66.5%)	68 (66.7%)	0.977
Smoking	150 (33.0%)	31 (30.4%)	0.606
Alcohol intake	21 (4.6%)	4 (3.9%)	0.757
BMI (kg/m^2^)	23.9 ± 2.5	24.7 ± 2.2	0.579
Heart rate (beats/min)	73.7 ± 14.1	77.7 ± 16.3	0.089
Systolic BP (mmHg)	138.5 ± 20.9	133.3 ± 24.0	0.071
Diastolic BP (mmHg)	77.2 ± 31.7	76.9 ± 12.8	0.742
GFR <60 ml/min/1.73 m^2^	20 (4.4%)	6 (5.9%)	0.523
**Medical history**
Hypertension	347 (76.4%)	74 (72.5%)	0.409
Dyslipidemia	311 (68.5%)	54 (52.9%)	0.003
Family history of CHD	19 (4.2%)	2 (2.0%)	0.287
**Laboratory examinations**
Fasting blood glucose (mmol/L)	7.76 ± 3.94	8.79 ± 2.73	0.784
HbA1c (%)	7.04 ± 1.50	7.06 ± 1.43	0.688
LDL (mmol/L)	2.60 ± 1.03	2.87 ± 1.07	0.306
HDL (mmol/L)	1.10 ± 0.56	1.10 ± 0.22	0.428
Triglycerides (mmol/L)	1.84 ± 1.94	1.93 ± 3.41	0.677
**Medical treatment**
ACEI	240 (53.0%)	68 (66.7%)	0.012
ARB	102 (22.5%)	9 (8.8%)	0.002
Beta blockers	379 (83.5%)	84 (82.4%)	0.783
Statins	449 (98.9%)	99 (97.1%)	0.159
CCB	194 (42.7%)	18 (17.6%)	<0.001
**Type of CHD**
Single-vessel	100 (22.0%)	6 (5.9%)	<0.001
Multi-vessel	354 (78.0%)	96 (94.1%)	<0.001
Diffuse	285 (62.8%)	43 (42.2%)	<0.001
**Stent implantation**
Stent number	2.85 ± 1.93	1.30 ± 0.95	<0.001
Total length (mm)	63.6 ± 40.7	31.2 ± 23.4	<0.001
Minimum diameter (mm)	2.69 ± 0.63	3.03 ± 4.34	0.048
**PCI**
Operation duration (min)	80.4 ± 34.7	60.7 ± 31.7	<0.001
Type (emergency)	137 (30.2%)	102 (100.0%)	<0.001

### Effects of FPG and HbA1c on the DES-ISR

**Figure 3:**
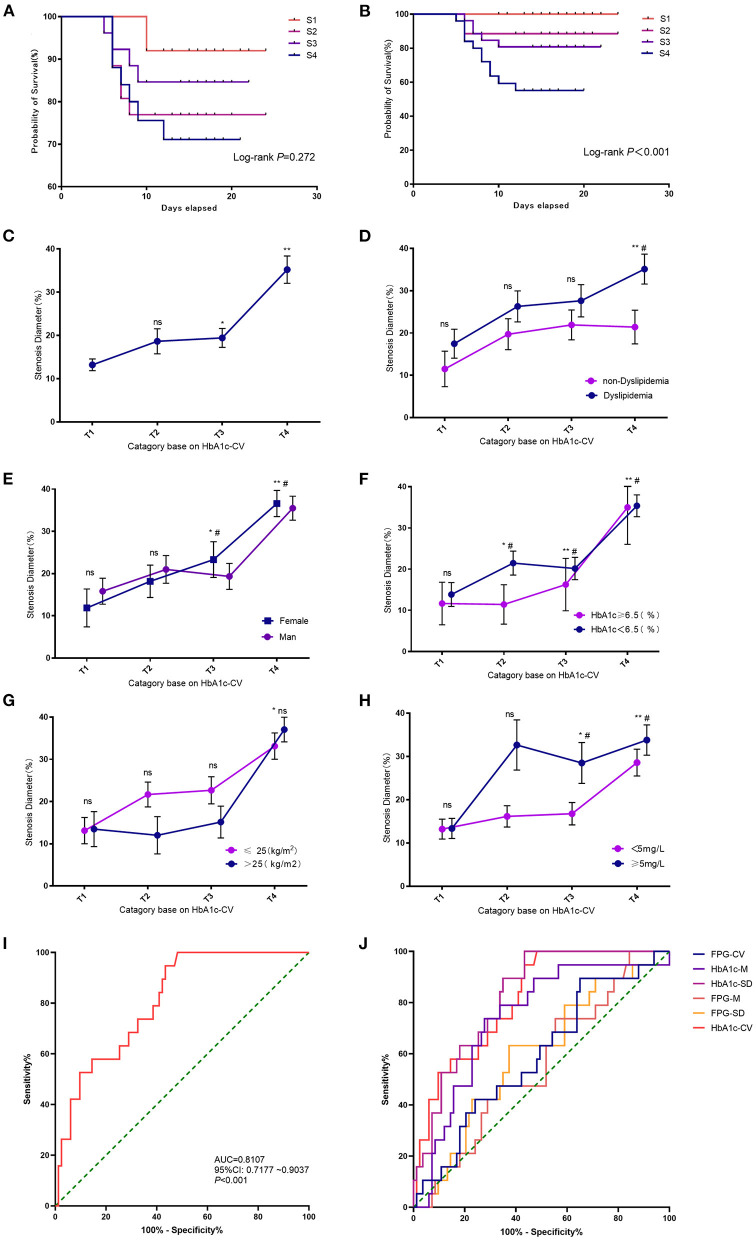
Effects of FPG and HbA1c on the DES-ISR. **(A)** Survival plot analysis between FPG-CV and the rate of DES-ISR. **(B)** Survival plot analysis between HbA1c and the rate of DES-ISR. **(C)** The rate of DES-ISR in the groups with different HbA1c-CV levels. **(D)** The rate of DES-ISR in patients with different HbA1c-CV levels as sub-grouped by dyslipidemia. **(E)** The rate of DES-ISR in patients with different HbA1c-CV levels as sub-grouped by gender. **(F)** The rate of DES-ISR in patients with different HbA1c-CV levels as sub-grouped by age. **(G)** The rate of DES-ISR in patients with different BMI as sub-grouped by HbA1c levels. **(H)** The rate of DES-ISR in patients with different CRP levels as sub-grouped by HbA1c-CV. **(I)** ROC curve and its predictive performance of HbA1c-CV. **(J)** ROC curve and the predictive performance of FPG-CV, HbA1c-M, HbA1c-SD, FPG-M, FPG-SD and HbA1c-CV. *P < 0.05, **P < 0.01 comparison among T1-T4 groups; ^#^*P* < 0.05 comparison between subgroups; ns = not significant.

In the validation cohort, the survival plot analysis showed that FPG- coefficient of variation (CV) exhibited no significant effect on the risk of DES-ISR (Figure 3A). High FPG-CV was not associated with the higher rate of DES-ISR (Supplementary Figure S2A). In the subgroup analysis, patients with dyslipidaemia at S4 group had higher rate of DES-ISR than that without dyslipidaemia (Supplementary Figure S2B). No significant differences were detected between groups stratified based on gender (Supplementary Figure S2C), HbA1c level (Supplementary Figure S2D) and body mass index (BMI; Supplementary Figure S2E). Patients with C-reactive protein (CRP) level ≥5 mg/L at S3 and S4 group had higher rate of DES-ISR than that with CRP level <5 mg/L (Supplementary Figure S2F).

HbA1c-CV was significantly associated with the risk of developing DES-ISR, and the higher levels of HbA1c-CV was correlated with higher rate of DES-ISR (Figures 3B,C). In the subgroup analysis, patients without dyslipidaemia at T4 group showed higher rate of DES-ISR than that with dyslipidaemia (Figure 3D). Female patients at T4 group had higher rate of DES-ISR when compared to male patients (Figure 3E). Patients with higher H1bA1c level also had higher rate of DES-ISR (Figure 3F). Patients with higher BMI tended to have higher rate of DES-ISR, but it was not statistically significant (Figure 3G). Patients with CRP ≥5 mg/L at T3 and T4 group had higher rate of DES-ISR than that with CRP <5 mg/L (Figure 3H). In ROC analysis, our results showed that HbA1c had better predictive ability for DES-ISR than HbA1c-M and HbA1c-SD. On other hand, FPG-CV, FPG-M and FPG-SD only showed fair diagnostic potential based on the ROC curve analysis (Figures 3I,J).

### Effects of C-Reactive Protein Level on the DES-ISR

**Figure 4:**
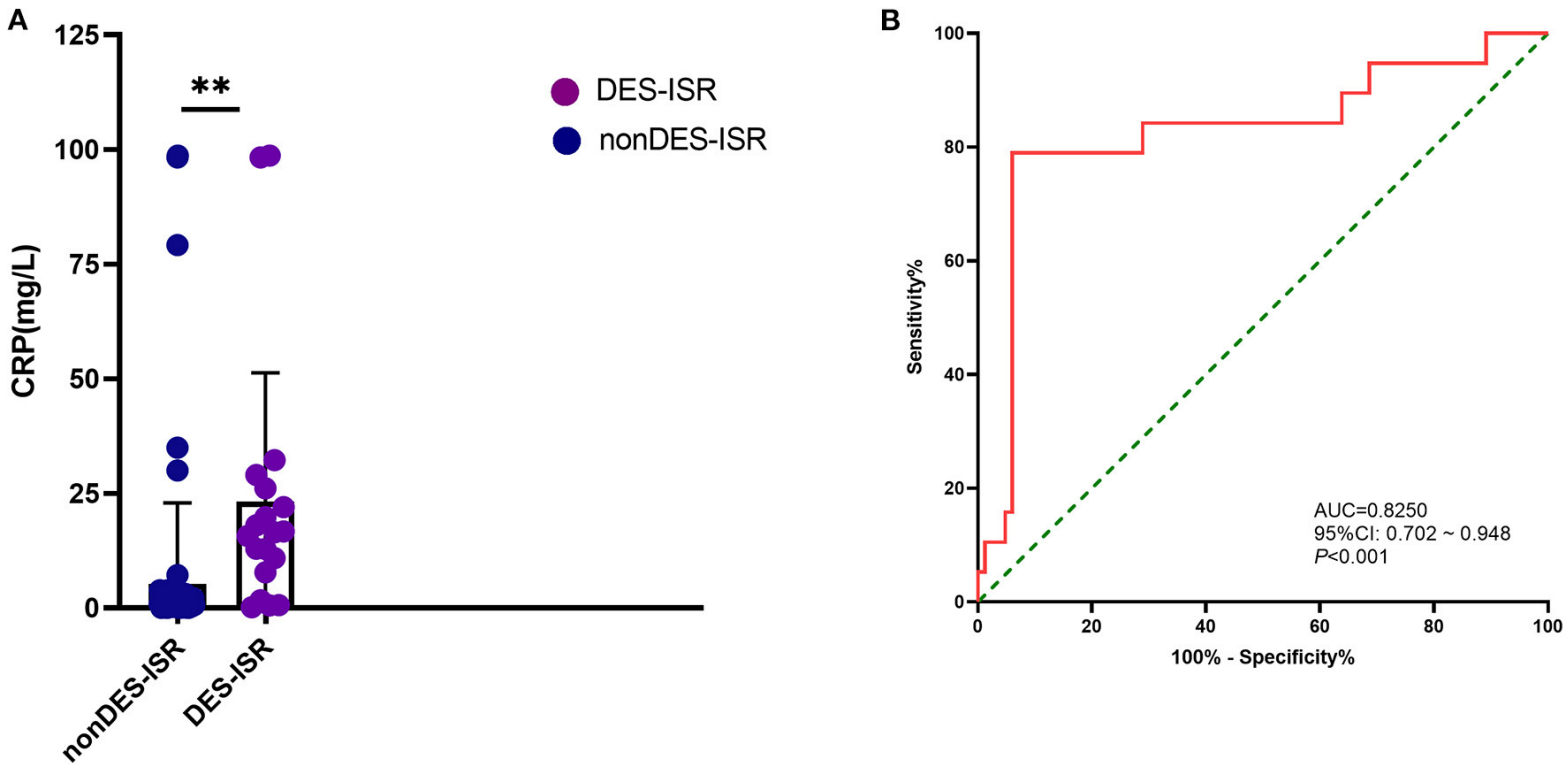
Effects of CRP level on the DES-ISR. **(A)** The expression levels of CRP in the non DES-ISR and DES-ISR group. **(B)** ROC curve and its predictive performed of CRP level. **P < 0.05.

**Figure 5:**
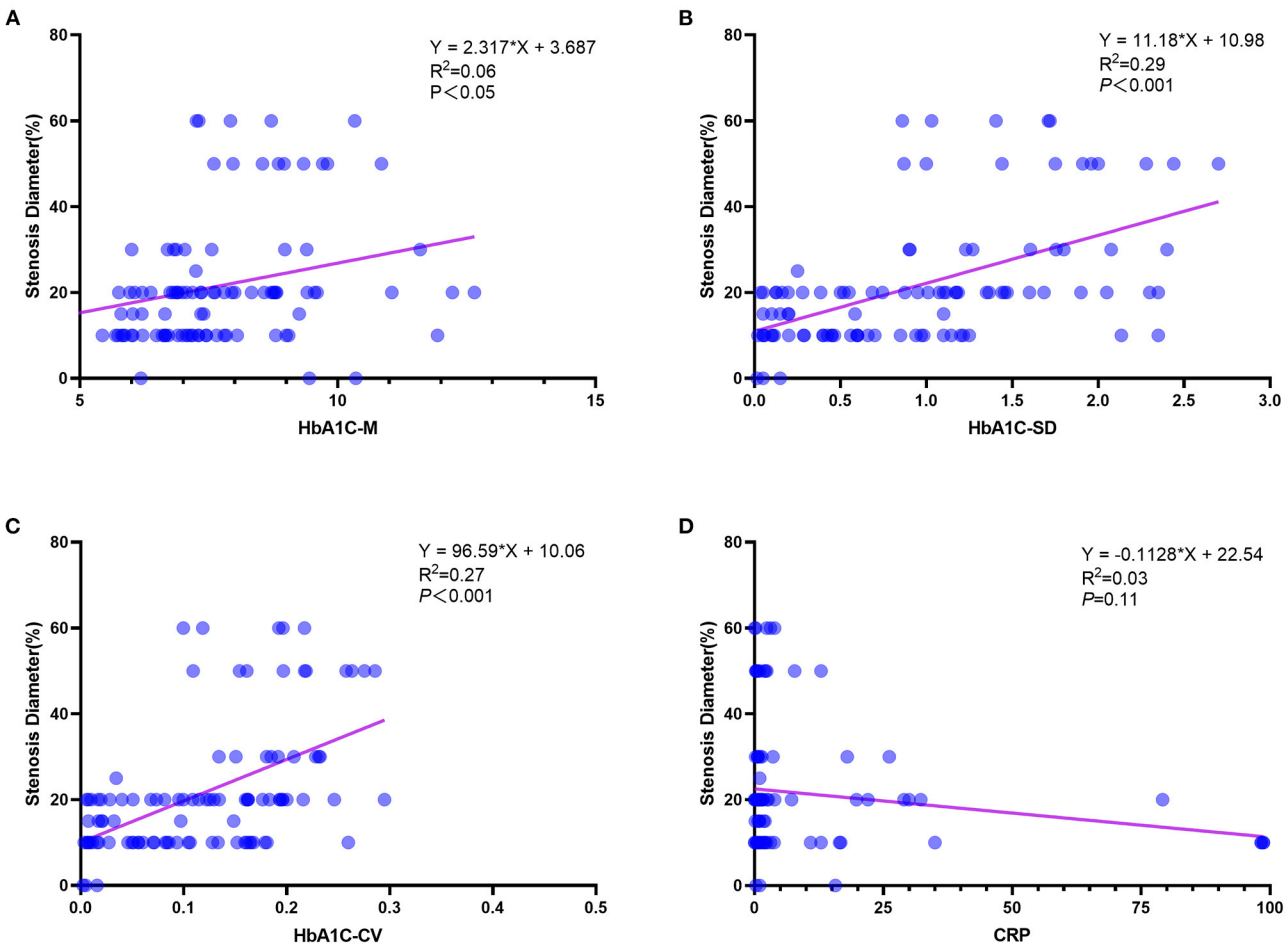
The association between different predictors and the rate of DES-ISR. **(A)** The correlation analysis between HbA1c-M and the rate of DES-ISR. **(B)** The correlation analysis between HbA1c-SD and the rate of DES-ISR. **(C)** The correlation analysis between HbA1c-CV and the rate of DES-ISR. **(D)** The correlation analysis between CRP and the rate of DES-ISR.

The diagnostic potential of CRP level was further assessed with ROC analysis. In the validation cohort, the CRP level was significantly higher in DES-ISR group than that in the non-DES-ISR group (Figure 4A). The ROC analysis showed that CRP had good predictive ability for DES-ISR (Figure 4B). The correlation analysis showed that HbA1c-M, HbA1c-SD and HbA1c-CV were positively correlated with the diameter of ISR (Figures 5A–C); while CRP was not significantly correlated with the diameter of ISR (Figure 5D).

### CATPCA Analysis of Clinical Variables in the Validation Cohort

**Figure 6:**
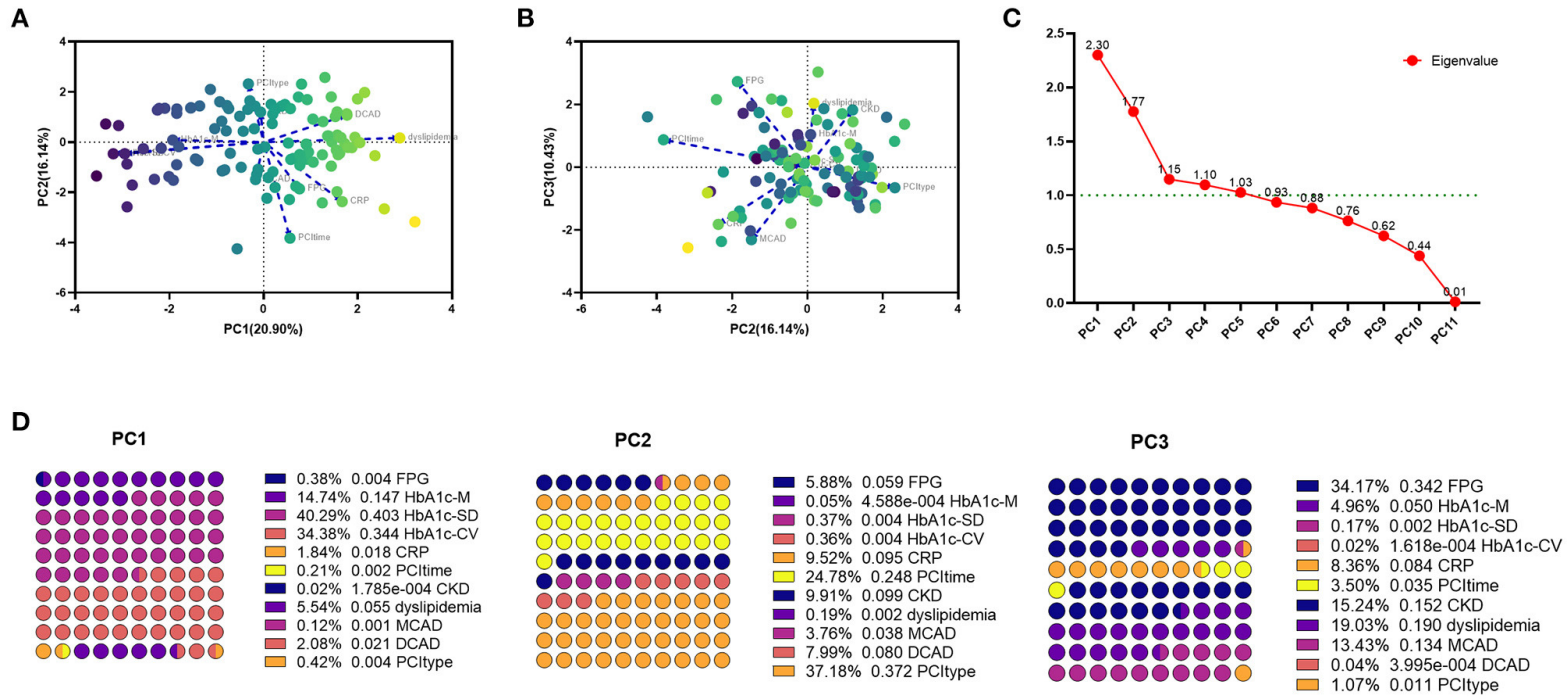
CATPCA analysis of clinical variables in the PSM cohort. **(A,B)** CATPCA biplot—component loadings of the most explanatory variables. **(C)** The scree plot of CATPCA. **(D)** Risk factors in PC1, PC2, and PC3.

Categorical principal component analysis of clinical variables was performed to determine important components for DES-ISR in the validation cohort. Based on CATPCA analysis, PC1, PC2, and PC3 explained 20.90, 16.14, and 10.43% of the total variation of the data of the validation cohort (Figures 6A–C). The most important components in PC1, PC2, and PC3 were illustrated in Figure 6D.

## Discussion

This case-control study retrospectively analyzed the clinical data of patients with coronary heart disease and diabetes. Our results showed that glomerular filtration rate <60 ml/min/1.73 m^2^, fasting blood glucose ≥6.5 mmol/L, multivessel coronary artery disease, coronary artery diffuse disease, percutaneous coronary intervention (PCI) operation time (≥60 min), and emergency PCI were associated with ISR. High Nomogram score was associated with the increased risk of ISR. Further analysis of the validation cohort showed that higher levels of HbA1c-coefficient of variation (CV) were significantly associated with the increased risk of ISR. HbA1c-CV exhibited good predictive ability for ISR in the validation cohort.

Compared with BMS, DES minimizes new intimal hyperplasia, and the coating on DES can cause delay in intimal hyperplasia. This intimal hyperplasia can last for many years. Therefore, the time when DES-ISR appears has been largely extended for several years after stent implantation (17). For DES, ISR remains a challenging clinical problem. The vascular stenosis caused by DES-ISR often requires revascularization of the target lesion. According to the US National Cardiovascular Data Registry, about 10% of PCI is used to treat ISR (18). The results of a domestic retrospective study showed that the treatment rate of DES-ISR revascularization in diabetic patients was 4.4% within 1 year and 7.7% within 2 years (15). Compared with the revascularization of primary coronary artery disease, although DES has been improved in recent years, the accumulation of DES-ISR therapy for many years (re-stent implantation or drug balloon) usually makes revascularization more severe and complex with worse prognosis when compared with the revascularization of primary coronary artery disease. DES treatment is currently the main treatment method for the treatment of ISR. Therefore, a proper prediction model to accurately identify the risk factors of DES-ISR in elderly diabetic patients can be helpful in optimizing the treatment and follow-up strategies, thus to reduce of TLR in the DES-ISR patients.

Studies have identified many factors that are associated with DES-ISR. Our analysis exhibited inconsistency with previous studies, which indicates that there are more accurate prediction methods for elderly diabetic patients, and the prediction model for non-specific populations is likely to miss important predictors for elderly diabetic patients. Age (<60 years) and diabetes have been confirmed to be important predictors of ISR (11). Our results showed that high FPG but not HbA1c level before stent implantation was associated with DES-ISR. Studies have pointed out that perioperative blood glucose control is more important in preventing DES-ISR (19). HbA1c reflects the long-term condition of blood glucose control. Therefore, HbA1c level immediately before surgery does not have an accurate predictive power. On the contrary, detecting HbA1c fluctuations may have better predictive value. Recent studies have pointed out that the high coefficient of variation (CV) of HbA1c is independently associated with DES-ISR (14). In the validation cohort, Nomogram can effectively predict the risk of DES-ISR within 2 years, but it showed no correlation with the degree of stenosis in the stent. We further analyzed the blood glucose variability indicators in the validation cohort and found that the hyperglycemia status during emergency PCI perioperative period and high HbA1c variability during follow-up were not only independent risk factors for DES-ISR, but also related to more severe stenosis.

The other risk factors screened out by the derivation cohort are consistent with the non-specific population prediction model (9). Multi-vessels and diffuse coronary artery disease (20), GFR < ml/min/1.73 m^2^ (21, 22) and so on are still risk factors for DES-ISR. Our analysis also paid attention to the correlation between operation-related factors (PCI operation time, etc.) and DES-ISR. Studies have pointed out that PCI adverse events (such as incomplete myocardial reperfusion), even under controlled conditions, operator variables are still significantly related to these adverse events, and differences between operators may also exist in the efficacy of PCI (23). Our results indicate that the continued collection of PCI operator-related factors to construct a data set to evaluate and improve the abilities and skills of individual operators.

Although the PCA results of the validation cohort and the CATPCA analysis results of the model generation cohort are not completely consistent, FPG and HbA1c variability are the main contributors to the main components. In this study, FPG-CV exhibited no significant effect on the risk of DES-ISR in the model cohort, and FPG-CV, FPG-M and FPG-SD only showed fair diagnostic potential based on the ROC curve analysis. However, previous studies have found that glucose control was associated with risk of DES-ISR. Ceriello et al. showed that insulin resistance was associated with ISR in patients undergoing coronary DES implantation at long-term angiographic follow-up (13). Zhao et al. (24) showed that elevated FPG level was correlated with higher restenosis risk in coronary heart disease patients underwent sirolimus-eluting stent implantation. Fibroblast growth factor-23 was positively correlated with FPG, and was of good value in predicting 2-year ISR risk (25). Moreover, a greater absolute change in FPG over 12 months post-PCI is an independent risk factor for 2- and 5-year MACE development in DES-implanted patients, especially in the diabetes and statin users (26). However, the Stent Restenosis and Metabolism (STREAM) study showed that addition of a single bedtime dose of insulin in patients with diabetes does not influence ISR (27). Therefore, further studies may be considered to consolidate the role of FPG in predicting DES-ISR. In terms of HbA1c, HbA1c-CV was significantly associated with the risk of developing DES-ISR, and the higher levels of HbA1c-CV was correlated with higher rate of DES-ISR; in addition, HbA1c-CV had better predictive ability for DES-ISR than HbA1c-M and HbA1c-SD, though these parameters all positive correlated with correlated with the diameter of ISR. In fact, high level of HbA1c-CV was associated with an increased risk of cardiovascular death (13), and recent studies showed that high HbA1c-CV was correlated with increased risk of ISR in patients with type 2 diabetes after stent implantation (14). These above results indicated that HbA1c may be an important factor in predicting the risk of DES-ISR. However, we should be cautious when interpreting the data regarding HbA1c, as we have not fully analyzed the other cofound factors such as gender, BMI and blood pressure, which has been associated with fluctuation of HbA1c (28).

Our findings should be interpreted in the context of following limitations. Firstly, this study is a retrospective analysis with small sample size, and all the enrolled patients were from a single center, and future investigation should consider larger sample size and multi-center studies. Secondly, we generated Nomogram chart based on the results of the multi-factor conditional logistic regression analysis, which provides an intuitive and convenient way to predict individualized predictions. However, there is a mutual conversion between categorical variable data and measurement data in the model, so it is possible weaken the interpretation of these risk factors for the final main result. Thirdly, multivariate analysis often requires a large sample of data, in order to give meaningful output, otherwise, the results are meaningless due to high variation of the data. Fourthly, CatPCA operates only on category indicator variables, and future studies may perform PCA analysis in continuous variables to reveal more significant findings from our current database. Fifthly, PCI technology development, especially the implant material, is an important factor affecting ISR, thus, we shall be cautious when interpreting our results, and our results still require further prospective cohort study or randomized controlled trials study to validate our conclusions.

## Conclusion

In conclusion, the present study showed that higher levels of HbA1c-CV group was significantly associated with the increased risk of ISR. HbA1c-CV exhibited good predictive ability for ISR in the validation cohort. FPG during the perioperative period of emergency PCI and HbA1c-CV during the follow-up period may represent key factors in predicting incidence of DES-ISR and the degree of stenosis. Thus, proper control of blood glucose fluctuations may beneficial in reducing the risk of DES-ISR. In the future studies, large sample size with multiple-center studies should be performed to consolidate the present findings.

## Data Availability Statement

The original contributions presented in the study are included in the article/Supplementary Material, further inquiries can be directed to the corresponding authors.

## Ethics Statement

The Institutional Review Boards at Shenzhen Sun Yat-sen Cardiovascular Hospital approved the present study. The patients/participants provided their written informed consent to participate in this study.

## Author Contributions

XK and QL conceived the project. MY and W-hT were responsible for the experimental design and application and writing the manuscript. SX performed data analysis for the manuscript. All authors read and approved the final manuscript.

## Funding

This study was supported by Guangdong Basic and Applied Basic Research Foundation (Nos. 2019A1515010329 and 2021A1515010178), Shenzhen Fundamental Research Program (No. JCYJ20180302173849459), the Fund of “Sanming” Project of Medicine in Shenzhen (No. SZSM201811096) and the Shenzhen Strategic Emerging Industry Development Special Fund (No. ZDYBH201900000007).

## Conflict of Interest

The authors declare that the research was conducted in the absence of any commercial or financial relationships that could be construed as a potential conflict of interest.

## Publisher's Note

All claims expressed in this article are solely those of the authors and do not necessarily represent those of their affiliated organizations, or those of the publisher, the editors and the reviewers. Any product that may be evaluated in this article, or claim that may be made by its manufacturer, is not guaranteed or endorsed by the publisher.
