# Supplementary material for: Investigation Into the Risk Factors Related to In-stent Restenosis in Elderly Patients With Coronary Heart Disease and Type 2 Diabetes Within 2 Years After the First Drug-Eluting Stent Implantation
Source: Front Cardiovasc Med. 2022 May 20;9:837330. doi: 10.3389/fcvm.2022.837330 (PMC9163371; doi:10.3389/fcvm.2022.837330)
Supplement: Supplementary file 1 [file Data_Sheet_1.pdf]

Supplementary Figure Caption

Supplemental Figure S1. The flow chart of the study design.

Supplemental Figure S2. Effects of FPG-CV on the DES-ISR. (A) The rate of DES-ISR in the groups with different FPG-CV levels. (B) The rate of DES-ISR in patients with different FPG-CV levels as sub-grouped by non-dyslipidemia and dyslipidemia. (C) The rate of DES-ISR in patients with different FPG-CV levels as sub-grouped by gender. (D) The rate of DES-ISR in patients with different BMI as sub-grouped by HbA1c levels. (E) The rate of DES-ISR in patients with different FPG-CV levels as sub-grouped by MBI. (F) The rate of DES-ISR in patients with different FPG-CV levels as sub-grouped by CRP levels. *$P < 0.05$, **$P < 0.01$ comparison among S1-S4 groups; $^{\#}P < 0.05$ comparison between subgroups; ns = not significant.
