# Supplementary figures and images for: Investigation Into the Risk Factors Related to In-stent Restenosis in Elderly Patients With Coronary Heart Disease and Type 2 Diabetes Within 2 Years After the First Drug-Eluting Stent Implantation
Source: Front Cardiovasc Med. 2022 May 20;9:837330. doi: 10.3389/fcvm.2022.837330 (PMC9163371; doi:10.3389/fcvm.2022.837330)

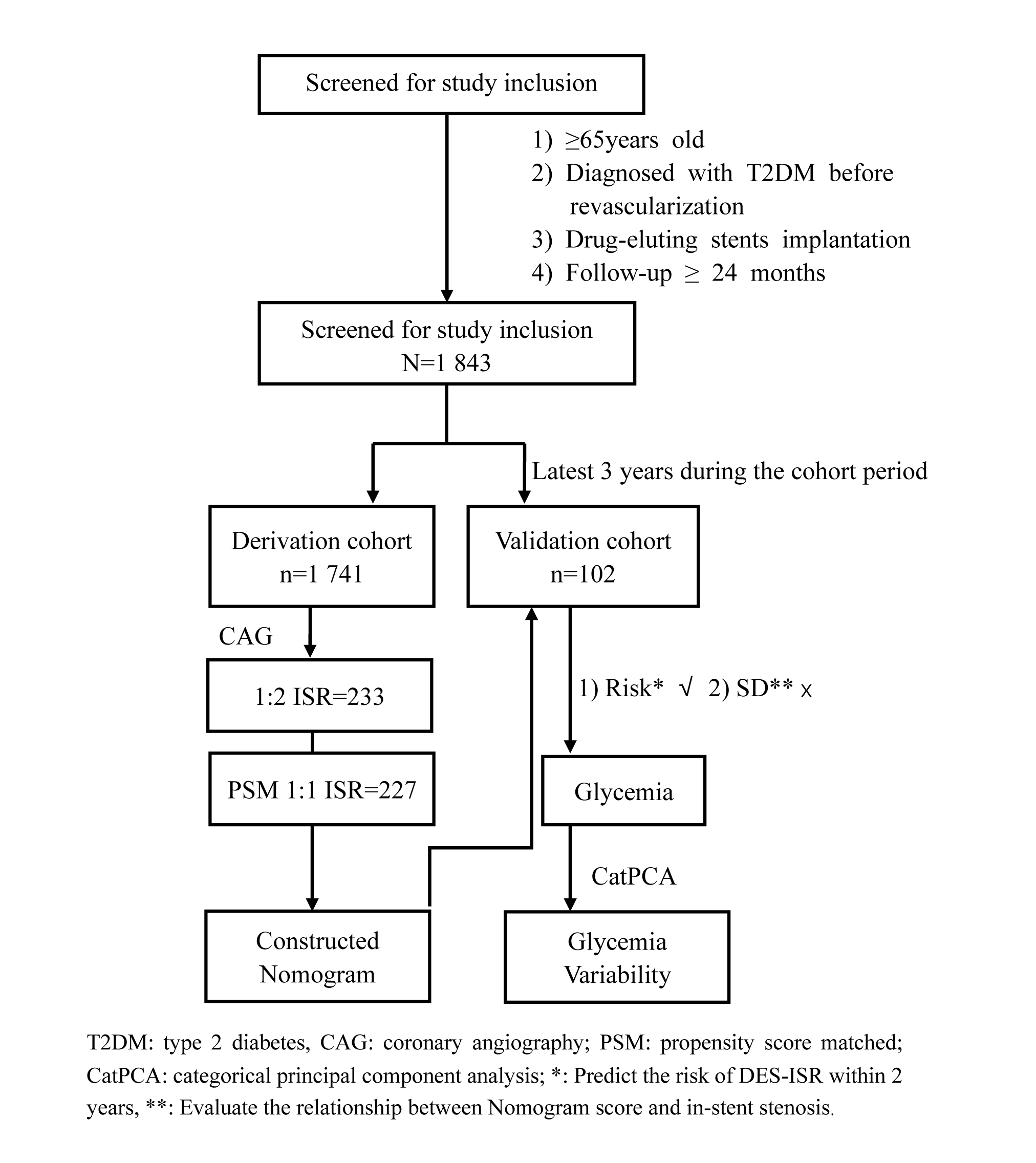

Supplement: Supplementary file 2 [file Image_1.TIF]

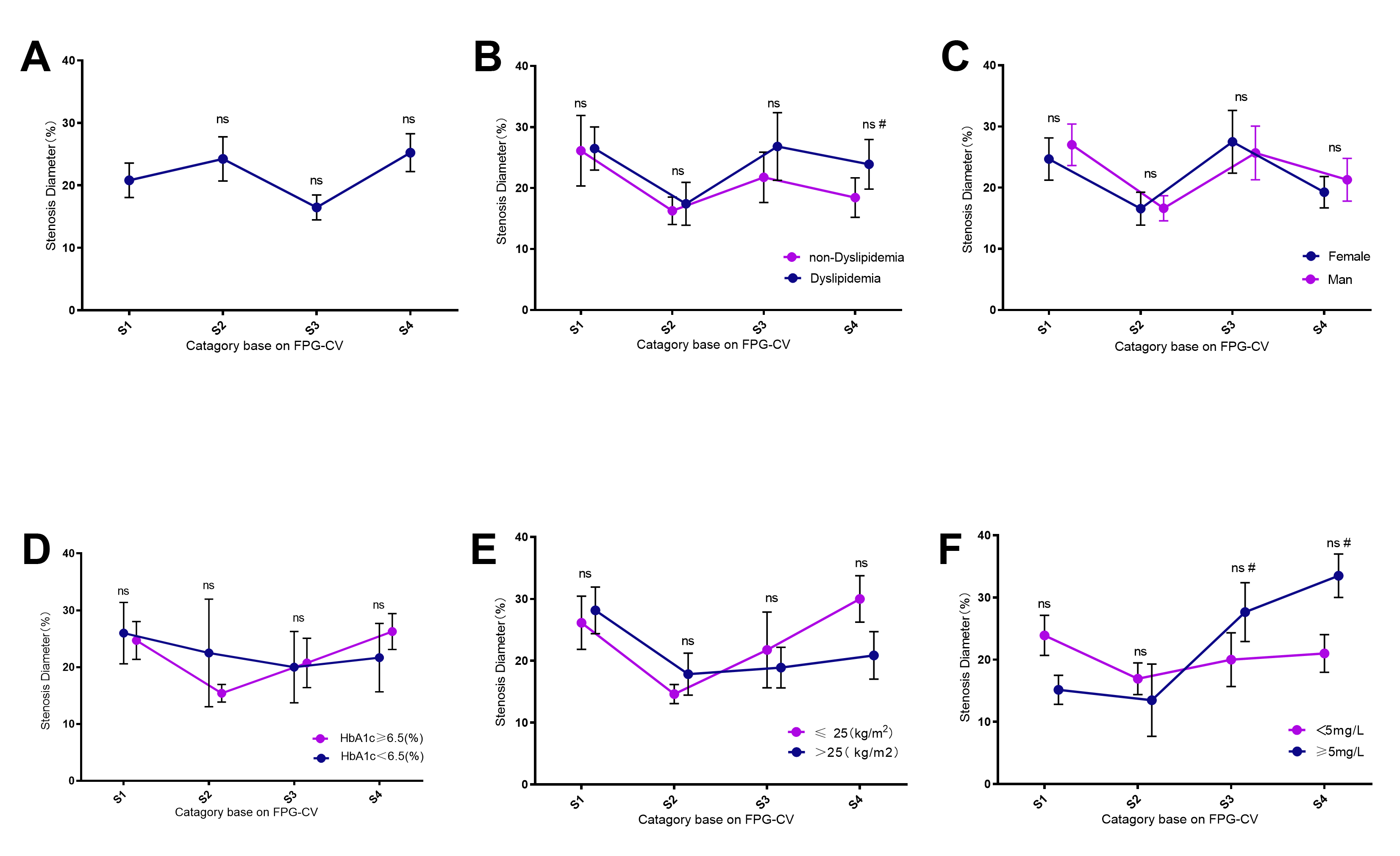

Supplement: Supplementary file 3 [file Image_2.TIF]
